# Cherubism in a 4-Year-Old Boy: A Clinical, Radiological, and Genetic Report

**DOI:** 10.1155/crid/9974548

**Published:** 2025-09-30

**Authors:** Menzile Seda Cosar, Kardelen Demirel, Ugur Baran Goz, Merih Seval Olmez

**Affiliations:** ^1^Department of Pediatric Dentistry, Hacettepe University Faculty of Dentistry, Ankara, Turkey; ^2^Department of Oral and Maxillofacial Surgery, Hacettepe University Faculty of Dentistry, Ankara, Turkey

**Keywords:** cherubism, computed tomography, familial fibrous dysplasia, giant cell granuloma, jaw expansion

## Abstract

Cherubism is a rare hereditary disorder, often referred to as familial fibrous dysplasia of the jaws, and is characterized by painless, bilaterally symmetrical enlargement of the facial structures. There are various clinical manifestations, ranging from the asymptomatic to the aggressive form. Since cherubism tends to regress spontaneously over time, therapeutic decisions are primarily guided by the severity of clinical presentation and individual patient needs. This case report highlights the clinical, radiological, and genetic findings of cherubism in a 4-year-old male patient.

## 1. Introduction

Cherubism (OMIM #118400) is a rare genetic disorder with autosomal dominant inheritance, typically causing painless, symmetrical swelling in the face. It is autoinflammatory in origin, and when limited to the craniofacial bones, it is referred to as familial fibrous dysplasia of the jaws [1–3]. The pathogenesis of cherubism has been linked to a heterozygous mutation affecting the SH3BP2 gene, which is located on chromosome 4p16 [2]. Cherubism progresses in three stages: expansion, stabilization, and regression. Cherubism typically begins during early childhood, often emerging in the second year of life and progressing until around the age of four. Following this phase, the condition tends to stabilize by approximately age seven and remains stable through the pubertal period [4, 5]. In some cases, regression occurs without treatment [6, 7].

Clinical findings show painless bilateral symmetrical enlargement of the maxilla and mandible. This often includes coronoid processes, but not condyles. In the early stages, submandibular lymph nodes can be affected, which may appear as palpable, nontender lymphadenopathy or mild swelling in the submandibular region [8]. The main reason for the name of “cherubic,” which is derived from Renaissance art, is the characteristic upward displacement of the eye globes and lower eyelid retraction [9]. Difficulty in chewing and speaking, sleep apnea, and visual impairment are among the functional consequences of cherubism [10, 11].

Dental findings, which may vary depending on the severity of the disease and the time of onset, include oligodontia, agenesis of teeth, malpositioned dentition, rudimentary molars, dental morphological anomalies, partial root resorption, and delayed or ectopic tooth eruption [12]. As a result of the mutation, disruption occurs in signaling pathways that regulate bone remodeling and immune system modulation. This disruption leads to increased inflammation and osteoclast production in the jawbones, resulting in the cyst-like radiolucent lesions observed in cherubism [13]. Radiographic imaging usually shows multilocular radiolucent areas with a soap-bubble appearance in the maxilla and mandible, while the mandibular condyles are not involved. The lesions show expansile remodeling and, as a result, subsequent thinning of the cortices [14]. If cortical perforation does occur, susceptibility to fractures may increase depending on the severity, and infection may develop [15]. Finally, nonneoplastic fibrous lesions and multinuclear giant cells have histologically been observed [5, 16]. This case report describes the clinical presentation, radiographic characteristics, and genetic mutation observed in a 4-year-old boy with cherubism.

## 2. Case Report

**Figure 1:**
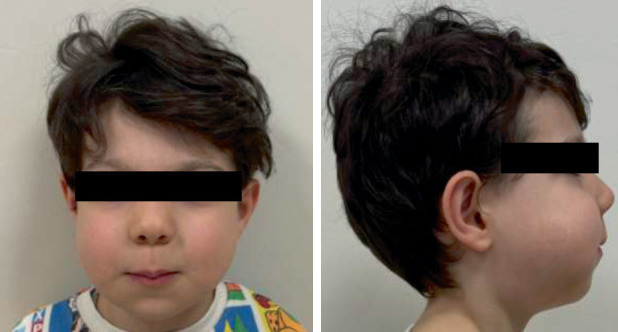
Bilateral diffuse extraoral swelling of the jaws.

**Figure 2:**
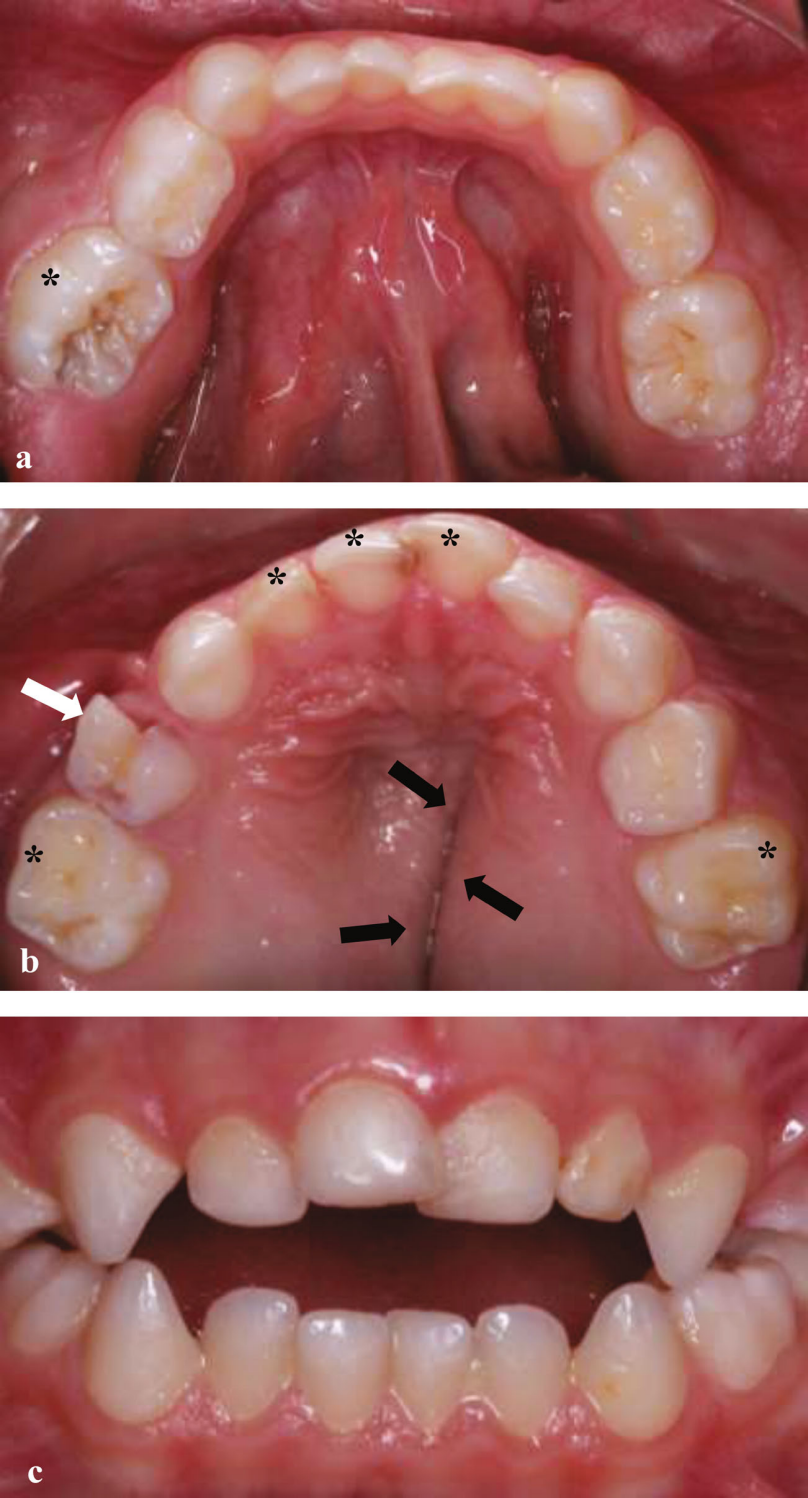
(a, b) Intraoral view of alveolar bone swelling, teeth with caries (black asterisk), maxillary right first primary molar with internal resorption (white arrow), incomplete cleft of the plate (black arrows), and (c) open bite.

A 4-year-old-boy was referred to the Department of Pediatric Dentistry following an initial evaluation by the Pediatric Oncology team, who suspected cherubism based on the presence of painless, symmetrical swelling affecting both the mandible and maxilla (Figure 1). Extraoral evaluation revealed bilateral jaw expansion, with no clinical evidence of lymphadenopathy. Although physical and cognitive development appeared normal, intraoral examination showed swelling of the alveolar bone, an open bite, and an incomplete cleft on the palate. Clinical examination revealed dental caries affecting the upper anterior teeth and primary molars. The maxillary left primary first molar also exhibited internal resorption, characterized by a pink spot (Figure 2).

A familial history was noted in the patient's 32-year-old mother, who experienced unilateral facial swelling on the left side during her childhood. The unilateral involvement observed in the mother, compared to the bilateral manifestation in the child, may be attributed to the variable expressivity of the SH3BP2 gene mutation. Due to limited health awareness within the family at the time, no medical consultation was sought during her early years. At the age of 15, a biopsy was performed, leading to a confirmed diagnosis of cherubism. Interestingly, she was treated and monitored by the same oncologist who is currently observing her child. The mother underwent denosumab therapy for 1 year. She currently remains asymptomatic and attends regular follow-up visits without the need for ongoing pharmacologic treatment.

**Figure 3:**
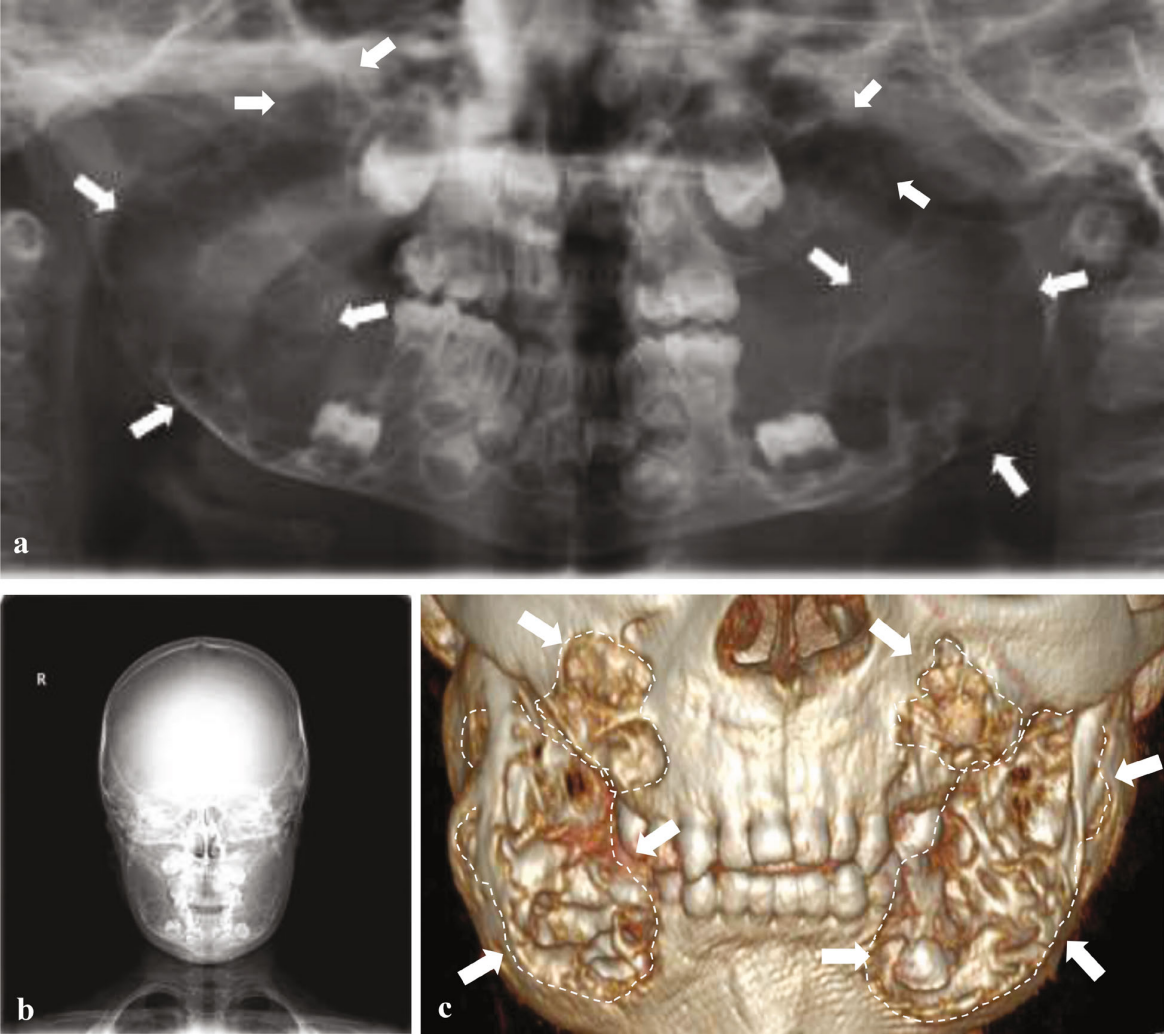
(a) Panoramic radiograph showing multilocular radiolucent lesions with a soap-bubble appearance involving both the maxilla and mandible (white arrows). (b) A skull radiograph showing multilocular radiolucency bilaterally in the right and left molar regions, in addition to thinning of buccal and lingual cortical plates. (c) Three-dimensional reconstructed CT image demonstrates expansile, multilocular radiolucent lesions involving the bilateral maxilla and mandible (white arrows), which are consistent with the characteristic fibro-osseous changes seen in cherubism.

In the panoramic radiograph, multilocular radiolucent lesions with a soap-bubble appearance were observed in the maxilla and mandible, sparing the mandibular condyles. The mandible, condyle, and coronoid processes were preserved in the lateral parts. The defined lesions widened the alveolar arch at the molar level (Figure 3a). A skull radiograph showed bilateral multilocular radiolucency in the right and left molar regions, and thinning of the buccal and lingual cortical plates (Figure 3b). Computed tomography was advised, and axial and coronal sections showed lesions reaching 6 × 3.5 cm in size at the most prominent location (Figure 3c). The lesions were characterized as osteolytic in nature, with an expansive, diffuse, and septated appearance.

**Figure 4:**
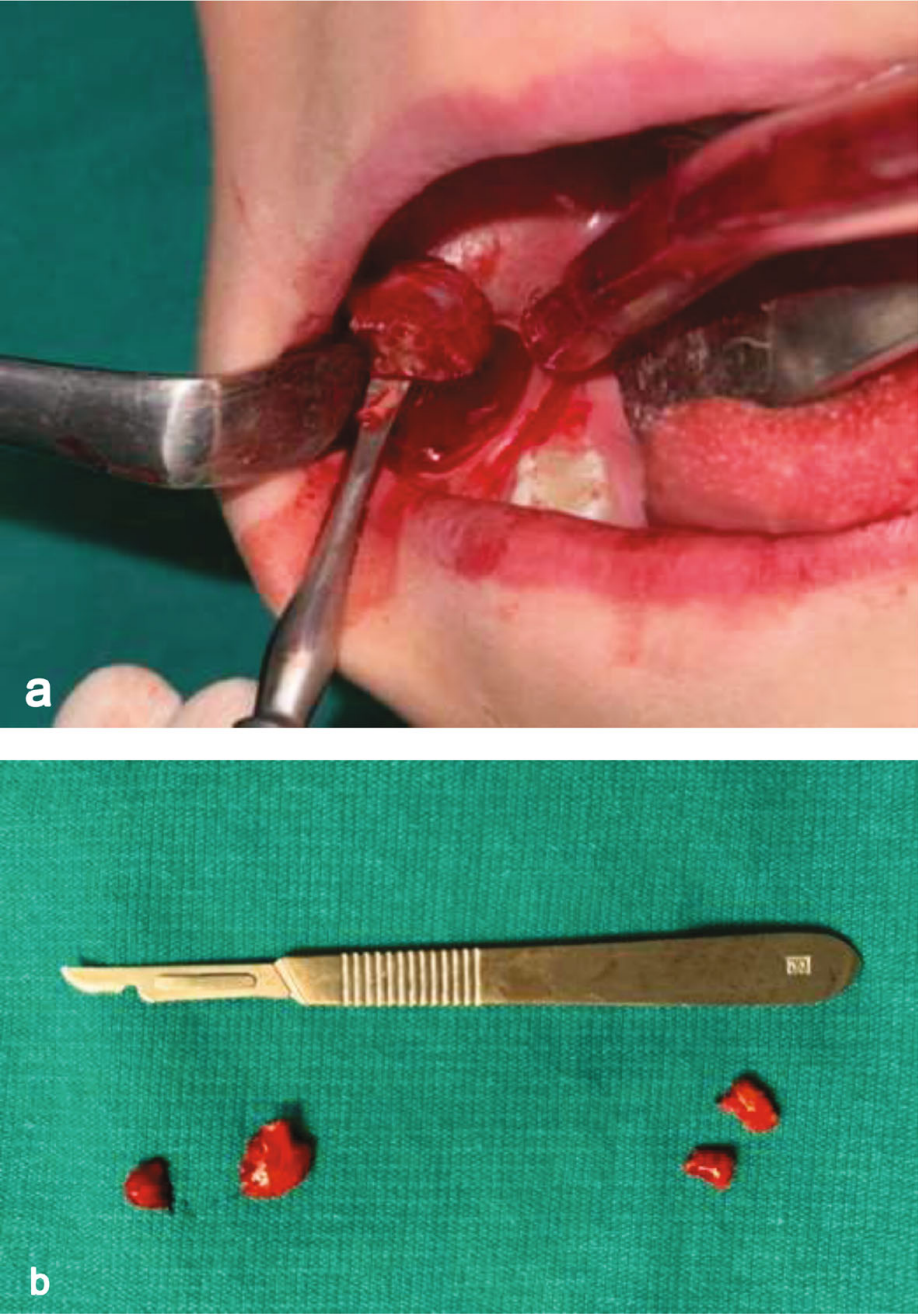
(a) Incisional biopsy. (b) Excised fragmented tissues.

Pediatric oncology recommended biopsy for differential diagnosis and initiation of treatment. Under general anesthesia, an intraoral mucosal incision was made in the bilateral mandibular molar region. Biopsy materials were collected from deep areas of the lesion, and biopsy specimens were sent to the pathology department for examination (Figure 4). In addition, the patient's decayed primary molars were restored, with the maxillary right primary first molar exhibiting internal resorption being extracted. In consideration of the patient's age, the carious proximal surfaces of the primary anterior teeth were flattened to create cleanable surfaces.

Microscopic sections of excised fragmented tissues showed a lesion consisting of monotonous spindle fibroblastic cells, with multinucleated giant cells being scattered among them, on a loose stromal ground rich in vascular structures that destroyed the bone tissue. Mitotic activity was not observed. Chronic inflammation rich in lymphocytes was observed in areas outside the lesion. In the biopsy report, it was stated that the findings were compatible with central giant cell granuloma. Correlation of clinical and genetic findings was found appropriate for differential diagnosis with cherubism.

Genetic evaluation showed c.1244G > A p. (Arg415Gln) (heterozygous) alteration in the SH3BP2 gene. This was classified as “pathogenic” in the Clinvar database, and the finding was found to be compatible with the diagnosis of cherubism.

**Figure 5:**
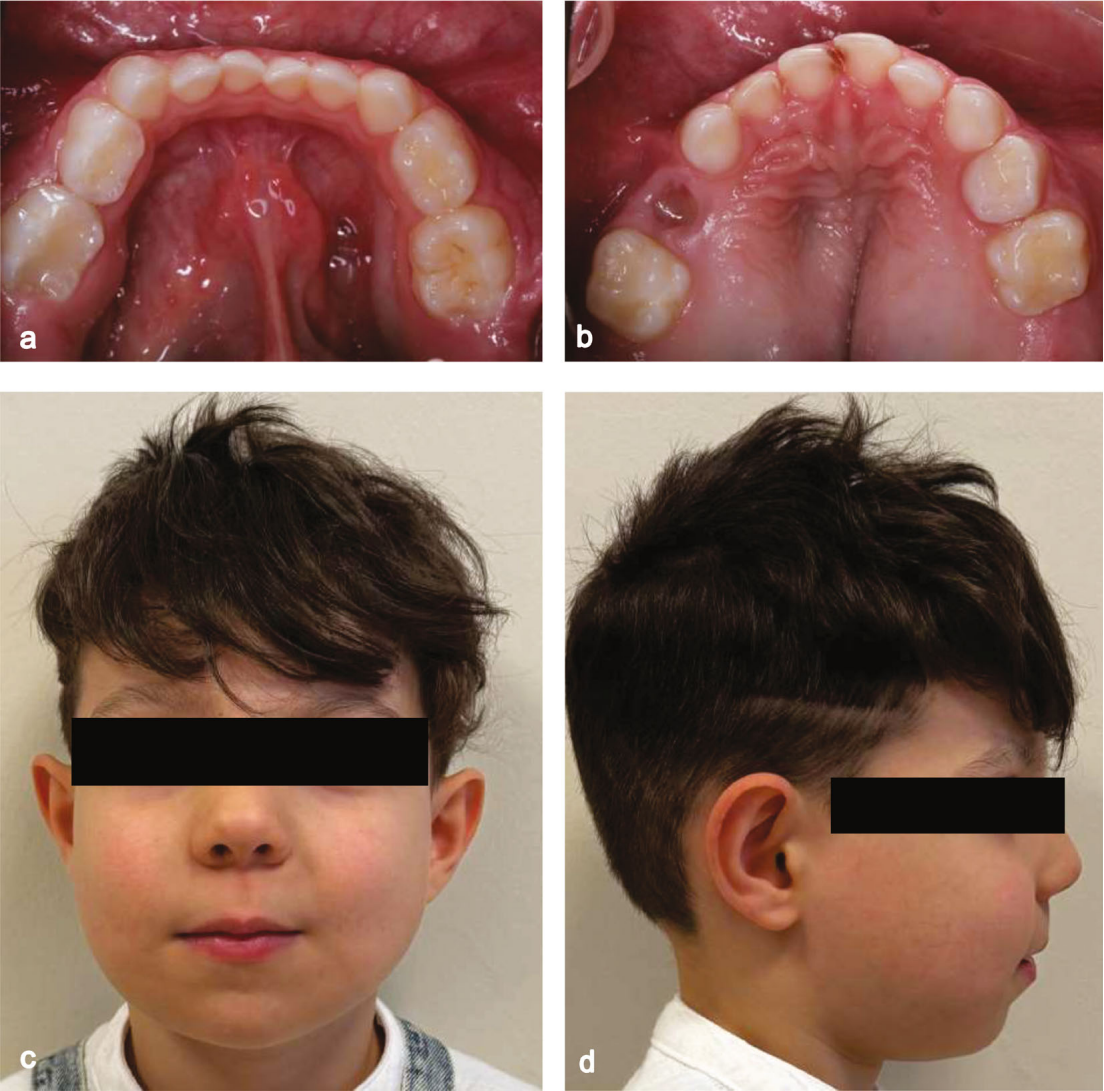
(a, b) Intraoral photographs at the 1-month follow-up. (c, d) Extraoral photographs at the 1-month follow-up.

In our case, after the diagnosis of cherubism was genetically confirmed, 50 mg denosumab, a human monoclonal antibody, was prescribed by the pediatric oncologist for therapeutic management. The main reason for initiating denosumab therapy was that the pediatric oncologist had observed that the same treatment for cherubism-related bone pathologies had been of benefit to the mother. The progression of the lesions has been monitored through regular recall appointments, with intraoral and extraoral photographs being taken in the 1-month follow-up. (Figure 5).

**Figure 6:**
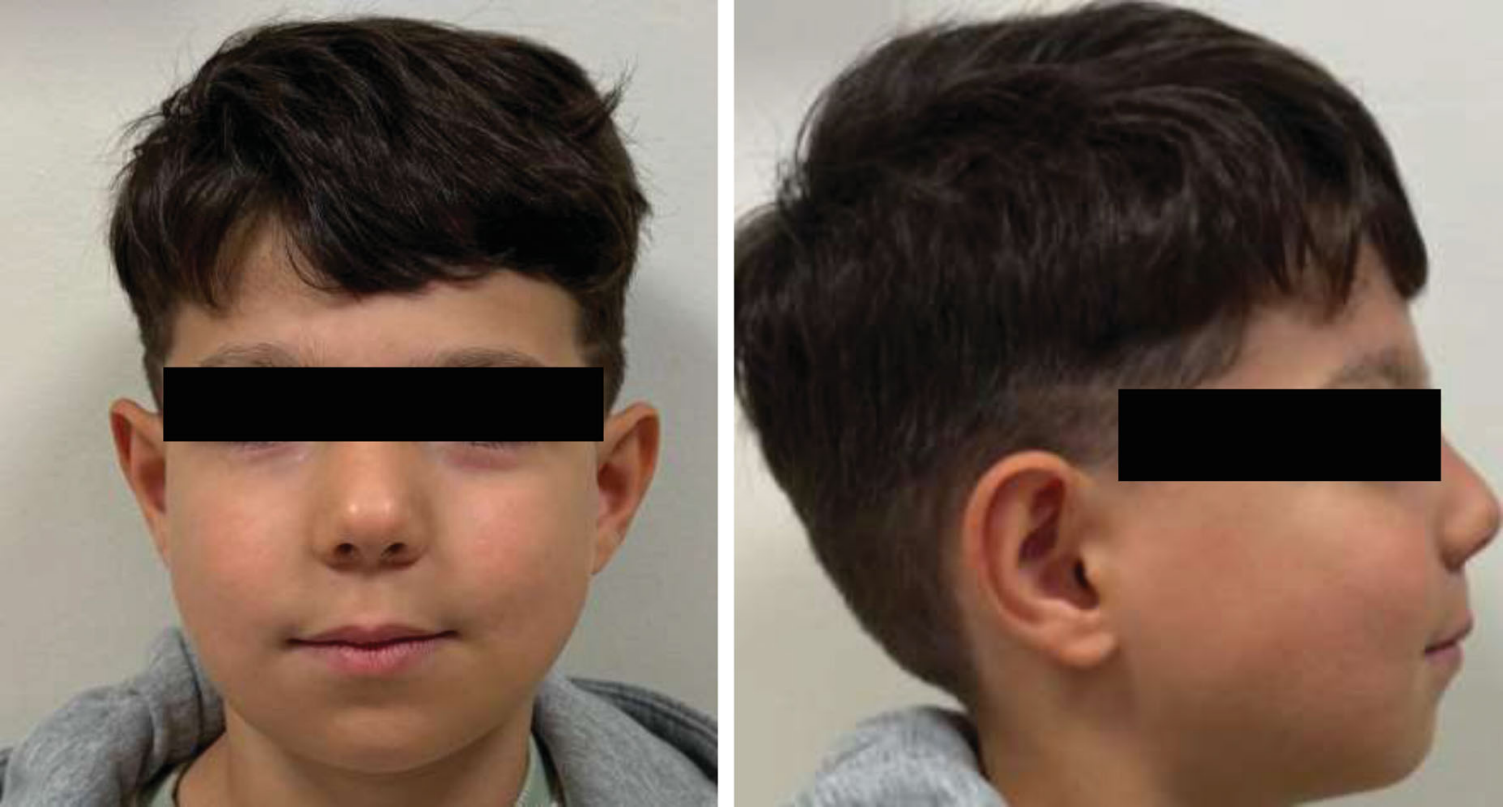
Extraoral clinical images demonstrating facial contours at the 6-month follow-up.

At the 6-month follow-up, the patient visited our clinic following a routine pediatric oncology check-up. The obtained paranasal sinus CT report indicated increased ossification within expansile fibro-osseous changes of the maxilla and mandible, with a slight reduction in the degree of expansion—more prominently in the mandibular rami. The transverse dimensions had decreased from 3.3 × 4.5 to 3.0 × 4.1 cm on the right side, and from 4.0 × 4.0 to 3.3 × 3.7 cm on the left side. The pediatric oncologist deemed the denosumab therapy sufficient and decided to discontinue the treatment. Extraoral photographs taken at the 6-month follow-up are presented (Figure 6).

**Figure 7:**
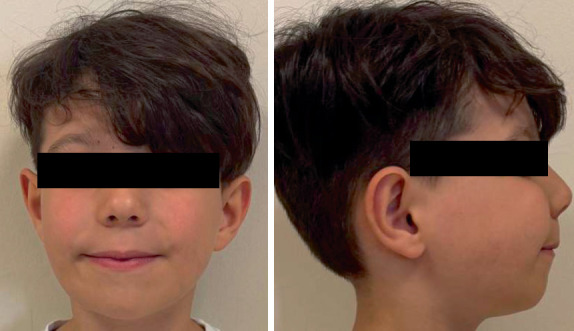
Extraoral clinical images demonstrating facial contours at the 12-month follow-up.

At the 12-month follow-up visit to our clinic—3 months after cessation of denosumab therapy—it was reported that the patient had developed hypercalcemia as a side effect of the treatment. According to the paranasal sinus CT scan performed at the 12-month oncology follow-up, increased ossification and reduced lytic changes were noted within the expansile fibro-osseous lesions of the mandible and maxilla. The mediolateral diameter of the lesion in the left posterior wall of the maxilla had decreased from 3.3 to 3.1 cm, and in the mandibular ramus on the left side from 3.3 to 2.9 cm. Extraoral photographs obtained at the 12-month follow-up are also presented (Figure 7).

## 3. Discussion

### 3.1. Clinical Features and Prognosis

Lesion spread in cherubism has been classified into six degrees: Grade I includes lesions found only in the mandible; Grade II involves both the mandible and maxilla; Grade III consists of aggressive lesions with root resorption in the mandible; Grade IV includes lesions with root resorption in both the mandible and maxilla; Grade V involves aggressive deformities including the coronoid and condyles along with both jaws; and Grade VI includes aggressive deformities affecting the orbitals along with the jaw involvement [6]. In the case report presented in this article, lesions are seen in both jaws, as is thus classified as Grade II. For mild cases, observational treatment involving annual clinical and radiographic examination is recommended, as lesions regress after puberty in most cases [6, 7, 17]. In more severe cases, surgery may be required for both functional and aesthetic management. If there is no serious problem, such as airway obstruction, surgical procedures should only be performed after puberty [18], as it has been reported that surgical shaping operations performed while the child is still growing can lead to tumor growth [19, 20].

### 3.2. Radiographic Features and Dental Findings

Multilocular lesions are radiologically evaluated using panoramic radiographs and computed tomography. Radiological imaging plays a role in the diagnosis of the disease and in monitoring its progression following surgical and therapeutic interventions [21].

The first radiographic signs of cherubism are radiolucent lesions located in the mandibular angle region. These asymptomatic lesions can affect the development or eruption of permanent teeth [22]. In addition to eruption problems, issues such as tooth displacement, root resorption, abnormally shaped teeth, early loss of primary teeth, missing teeth, and various malocclusions can be found and may require treatment [23–25]. A systematic review published in 2022 has reported that 30% of cases involved tooth loss, 15% involved tooth displacement, and only 2% had no tooth involvement [10].

If there is airway obstruction and the tongue is positioned backward, various respiratory problems, such as mouth breathing, obstructive sleep apnea, and chronic infections, can be observed [11, 26].

### 3.3. Differential Diagnosis

Cherubism is classified among nonneoplastic bone disorders primarily affecting the jaws [27]. The differential diagnosis includes several conditions, such as central giant cell granuloma, giant cell tumor, fibrous dysplasia, and brown tumors associated with hyperparathyroidism [10, 28]. Except for secondary hyperparathyroidism, hyperparathyroidism is rarely seen in children and can be distinguished from cherubism through blood analysis. In cherubism, serum calcium levels are normal, parathyroid hormone (PTH) levels are elevated, and phosphorus levels are low [22]. Moreover, mutations reported in many genes that are associated with hyperparathyroidism, such as CDC73 and CASR, are not related to the SH3BP2 gene [29].

Fibrous dysplasia is associated with a missense mutation in the GNAS1 gene on Chromosome 20 and is nonhereditary. In addition, the asymmetrically occurring lesions more commonly affect the maxilla and cherubic facial appearance is not present [16].

In central giant cell granuloma, which has histopathological features similar to cherubism, the lesions are most often located in the anterior region of the mandible, localized, and are not symmetrical. The fact that the lesions are acquired and the SH3BP2 mutation is not detected in genetic testing is important for differentiating the condition during diagnosis [16].

In addition to clinical, radiological, and histopathological assessments, genetic analysis plays a crucial role in the differential diagnosis of cherubism [30]. This condition is diagnosed through mutation of the SH3BP2 gene, a regulatory protein in immune response signaling, located on chromosome *4p16* [31]. Due to these mutations, the increase in bone resorption by osteoclasts leads to the formation of expansive lytic lesions in the jaws [2, 32]. The definitive diagnosis was likewise in our case made genetically.

### 3.4. Management and Treatment

Cherubism is typically a self-limiting condition, with spontaneous regression of the tumor-like lesions often occurring after puberty [22]. Consequently, the standard approach to management involves long-term monitoring, particularly in cases with mild clinical presentation. Consistent with this, the majority of reported cases have favored a conservative “wait and see” strategy, especially when the disease exhibits a less aggressive course [3]. Early surgical intervention is generally discouraged, as it has been associated with an increased risk of recurrence [33]. Indeed, several cases have documented lesion regrowth following minor surgical procedures [34–37].

In cases where the disease remains low-grade and is not accompanied by functional or structural complications, surgical treatment is often deemed unnecessary [3]. However, for more aggressive forms, procedures such as enucleation or curettage may be warranted after puberty to alleviate maxillofacial deformities and prevent further progression, thereby improving outcomes [38]. Surgical recontouring may also be indicated in situations involving significant aesthetic or functional impairments—such as nasal obstruction, proptosis, disfigurement with psychosocial impact, speech impairment, or difficulties in mastication and swallowing [22, 33].

Although delayed intervention until after puberty is the general principle, certain cases may require earlier therapeutic measures, including surgical or pharmacological approaches [3]. Various pharmacologic agents have been explored in the literature for their potential efficacy in managing cherubism lesions, including bisphosphonates, calcitonin, corticosteroids, denosumab, imatinib, interferon, and tumor necrosis factor (TNF) inhibitors [3].

In this case, denosumab was used for the therapeutic treatment of cherubism. Denosumab is a human monoclonal antibody that exerts its therapeutic effects by binding to and inhibiting the receptor activator of nuclear factor kappa-B ligand (RANKL), thereby suppressing osteoclast differentiation and function. The antibody is primarily approved for the treatment of conditions characterized by increased osteoclastic activity, such as osteoporosis, giant cell tumors of bone, and bone metastases with associated lytic lesions [39, 40]. Beyond its approved indications, denosumab has also been employed off-label in the management of osteoclast-mediated bone pathologies, including fibrous dysplasia, central giant cell granuloma, and cherubism [41, 42].

In the context of cherubism, denosumab has shown potential as a therapeutic option in cases unresponsive to conventional treatment. For example, Kugushev et al. reported the use of denosumab in a 9-year-old patient who had not responded to bisphosphonate therapy [43]. Following 6 months of denosumab treatment, a notable increase in bone density and a reduction in the size of the tumor-like lesions were observed, suggesting that denosumab could be effective in modulating disease progression through osteoclast inhibition.

In our case, denosumab therapy was discontinued after 6 months, as the pediatric oncologist considered the administered dose to be sufficient based on clinical and radiological improvements. However, at the 12-month follow-up—3 months after cessation of treatment—the patient developed hypercalcemia, a known rebound effect associated with denosumab withdrawal, particularly in pediatric populations. This adverse event aligns with previously reported cases in the literature, where rebound hypercalcemia has been observed following the discontinuation of denosumab in children [44, 45]. Although denosumab has demonstrated therapeutic potential in the management of cherubism and other osteoclast-mediated conditions, such findings underscore the importance of vigilant post-treatment metabolic monitoring as pediatric patients may be particularly susceptible to calcium imbalance due to increased bone turnover after cessation of therapy.

## 4. Conclusion

Cherubism, although rare, is a fibro-osseous disease that emerges during childhood and can be diagnosed through clinical, radiographic, histological, and genetic observation. If certain signs, such as bilateral swelling in the jaws, eruption anomalies, or displacement or absence of teeth, are encountered, the possibility of cherubism should be considered. In young patients with multilocular radiolucent lesions, it is crucial to distinguish cherubism from other lesions with similar clinical and radiological features, such as fibrous dysplasia or central giant cell granuloma. Early and accurate diagnosis ensures correct referral to the appropriate departments, as well as facilitating effective treatment and follow-up protocols through a multidisciplinary approach. Considering the potential for spontaneous regression of the disease during the growth and development period in children, it is important that unnecessary surgical interventions are avoided and that individualized follow-up protocols are created. Therefore, it is essential to raise awareness among the dentists who play a significant role in the diagnosing and monitoring of the jaw lesions associated with genetic conditions, such as cherubism.

## Data Availability

The data that support the findings of this study are available on request from the corresponding author. The data are not publicly available due to privacy or ethical restrictions.
